# First Principles Prediction of the Magnetic Properties of Fe-*X*_6_ (*X* = S, C, N, O, F) Doped Monolayer MoS_2_

**DOI:** 10.1038/srep03987

**Published:** 2014-02-05

**Authors:** Nan Feng, Wenbo Mi, Yingchun Cheng, Zaibing Guo, Udo Schwingenschlögl, Haili Bai

**Affiliations:** 1 Tianjin Key Laboratory of Low Dimensional Materials Physics and Preparation Technology, Institute of Advanced Materials Physics, Faculty of Science, Tianjin University, Tianjin 300072, China; 2 PSE Division, King Abdullah University of Science and Technology (KAUST), Thuwal 23955-6900, Saudi Arabia; 3 Core Laboratories, King Abdullah University of Science and Technology (KAUST), Thuwal 23955-6900, Saudi Arabia

## Abstract

Using first-principles calculations, we have investigated the electronic structure and magnetic properties of Fe-*X*_6_ clusters (*X* = S, C, N, O, and F) incorporated in 4 × 4 monolayer MoS_2_, where a Mo atom is substituted by Fe and its nearest S atoms are substituted by C, N, O, and F. Single Fe and Fe-F_6_ substituions make the system display half-metallic properties, Fe-C_6_ and Fe-N_6_ substitutions lead to a spin gapless semiconducting behavior, and Fe-O_6_ doped monolayer MoS_2_ is semiconducting. Magnetic moments of 1.93, 1.45, 3.18, 2.08, and 2.21 μ_B_ are obtained for *X* = S, C, N, O, and F, respectively. The different electronic and magnetic characters originate from hybridization between the *X* and Fe/Mo atoms. Our results suggest that cluster doping can be an efficient strategy for exploring two-dimensional diluted magnetic semiconductors.

As a layered transition-metal dichalcogenide semiconductor, MoS_2_ has attracted considerable attention for its distinctive electronic, optical, and catalytic properties^1^,^2^,^3^. It consists of stacked S-Mo-S monolayers with weak van der Waals interaction and can be mechanically exfoliated to atomically thin sheets^4^. The 1.2 eV indirect band gap of bulk MoS_2_ (*D*_6*h*_ point group) transforms into a 1.8 eV direct gap for monolayer MoS_2_ (*D*_3*h*_ point group). An S-Mo-S monolayer consists of a hexagonal plane of Mo atoms sandwiched between two hexagonal planes of S atoms. These planes are bonded covalently, with the S atoms in a trigonal prismatic arrangement. Because monolayer MoS_2_ has a high thermal stability, no dangling bonds, and an intrinsic direct band gap, it can be designed for switching device applications. Especially, a field-effect transistor based on monolayer MoS_2_ has shown a high mobility of 200 cm^2^/(Vs), a current on/off ratio of 10^8^, and an ultralow standby power dissipation^4^. The spin-orbit coupling and absence of inversion symmetry induce spin splitting at the valence band (VB) maximum and, therefore, suppress the spin relaxation to enhance the spin lifetime^5^,^6^. Consequently, monolayer MoS_2_ is a promising material for spintronics and nanoelectronic devices.

Dilute magnetic semiconductors (DMSs) have been in the focus of extensive researchwith a significant amount of theoretical and experimental efforts directed to transition metal doped III–V and II–VI three-dimensional systems^7^,^8^,^9^,^10^. For future spintronics devices, the development of two-dimensional DMSs is very important, because of the possibility to control both the electrical and magnetic properties by tuning the carrier density^11^. Recently, the formation of a two-dimensional DMS has been proposed for monolayer MoS_2_ doped by Mn, Fe, Co, and Zn^12^,^13^. It is possible to change the carrier type in MoS_2_ by substition of Mo by other metal atoms^14^. However, single atom doping is difficult to achieve when the doped atoms can form clusters due to a strong thermodynamic driving force^15^. In addition, S can be substituted by other nonmetals (F, Cl, and Br), which will induce *n*-doping and magnetism. Recently, it has been demonstrated that Fe-N_4_and Fe-C_4_ clusters doped in graphene lead to long-range ferromagnetism by carrier mediation^16^. It is expected that Fe-nonmetal doping can be applied to monolayer MoS_2_ to tailor the charge and magnetic states.

We investigate the effect of Fe-*X*_6_ doping on the electronic structure and magnetic properties of monolayer MoS_2_ by replacing one Mo atom by Fe, which is surrounded by various combinations of S, C, N, O, and F. We find that single Fe and Fe-F_6_ substitutions transform semiconducting monolayer MoS_2_ into a half-metal, that Fe-C_6_ and Fe-N_6_ doping result inspin gapless semiconductors, and that Fe-O_6_ doped monolayer MoS_2_ retains the original semiconducting properties. Total magnetic moments of 1.93, 1.45, 3.18, 2.08, and 2.21 μ_B_ per 4 × 4 × 1 supercell are induced in Fe-S_6_, Fe-C_6_, Fe-N_6_, Fe-O_6_, and Fe-F_6_ doped monolayer MoS_2_, respectively.

## Calculation details

**Figure 1:**
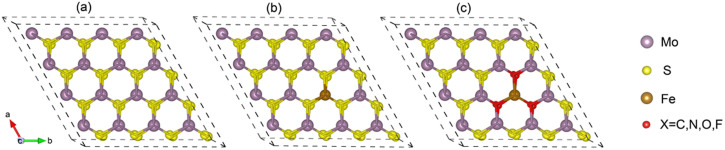
Structure of the supercell used for the calculations: (a) Pristine MoS_2_. (b) Fe-S_6_ and (c) Fe-*X*_6_ (*X* = C, N, O, F) clusters in monolayer MoS_2_. The purple, yellow, gold, and red spheres represent Mo, S, Fe, and *X* atoms, respectively.

Our first principles calculations are based on density functional theory^17^ and the projector augmented wave method^18^ as implemented in the Vienna Ab initio Simulation Package^19^. The Perdew-Burke-Ernzerhof^20^ spin-polarized generalized gradient approximation is used for the exchange-correlation potential and the plane-wave cutoff is set to 544 eV. We use a 4 × 4 × 1 MoS_2_ supercell with a vacuum region of 10 Å, as shown in Fig. 1. The Brillouin-zone integrations are performed on a 4 × 4 × 1 *k* mesh. All structures are fully optimized until the force on each atom is less than 0.01 eV/Å and the total energy converged to 10^−5^ eV. In order to illustrate the nature of the charge transfer, we calculate the difference between the valence electron densities of the Fe-*X*_6_ doped systems and the corresponding free atoms.

## Results and discussion

The symmetry of monolayer MoS_2_ with a Mo vacancy remains *C*_3*v*_^13^, which is also valid for Fe-*X*_6_ cluster doping. Under the structure relaxation the symmetry is maintained, since the distances between the Fe and six nearest *X* atoms are exactly the same. Table 1 gives the distances between the *X* and Fe (*d*_Fe-*X*_) or Mo (*d*_Mo-*X*_) atoms, and *X*-Fe-*X* bond angles for the Fe-*X*_6_ doped systems. The Fe-*X* bond lengths are 2.29, 1.93, 1.98, 2.15, and 2.18 Å for *X* = S, C, N, O, and F, respectively, which is smaller than the bulk Mo-S distance (2.41 Å), whereas the bond lengths between the *X* and nearest Mo atoms are 2.42, 2.04, 2.04, 2.03, and 2.21 Å. For pristine monolayer MoS_2_, the band gap of 1.7 eV is consistent with previous theoretical studies^12^,^21^,^22^ and photoluminescence results^23^. Strong hybridization appears between the Mo *d* and S *p* states in both the VB and conduction band (CB). Furthermore, the VB maximum and CB minimum are governed by the Mo *d* orbital.

**Figure 2:**
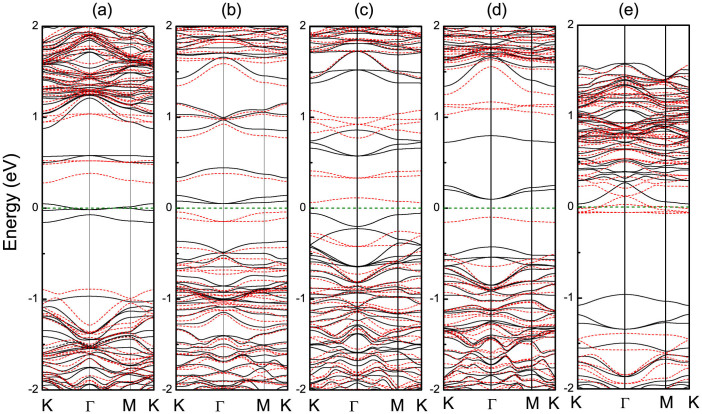
Electronic band structures for (a) Fe-S_6_, (b) Fe-C_6_, (c) Fe-N_6_, (d) Fe-O_6_, and (e) Fe-F_6_ doping. The black solid and red dashed lines denote the spin-up and spin-down channels, respectively. The Fermi level is indicated by a horizontal olive dashed line.

The band structure of Fe-*X*_6_ doped monolayer MoS_2_ is plotted in Fig. 2. Energy level splitting occurs between the spin-up and spin-down channels near the Fermi level, which induces magnetic moments. For Fe-doped MoS_2_ the spin-up CB minimum shifts down across the Fermi level and yields metallicity, while the spin-down channel remains semiconducting with a gap of 1.18 eV, indicating that the Fe-S_6_ doped system is half-metallic. To test whether the half-metallic character is sensitive to the concentration of the dopant, we have calculated the band structure of a 5 × 5 × 1 supercell, corresponding to a reduced concentration, and observe no relevant changes. The Fe-C_6_ doped system shows a semiconducting character for each spin channel with band gaps of 0.40 and 0.32 eV for the spin-up and spin-down channels, respectively. The gap between the spin-up VB and spin-down CB is only 0.08 eV wide. Wang^24^ and Hu^25^ use the term “gapless” for an energy gap that is smaller than 0.1 eV. In this sense, Fe-C_6_ doped monolayer MoS_2_ is a spin gapless semiconductor. In the case of Fe-N_6_ doping the system stays semiconducting with band gaps of 0.62 and 0.33 eV for the spin-up and spin-down channels, respectively. Since the gap between the spin-up CB and spin-down VB is 0.11 eV, as shown in Fig. 2(d), the system is also a spin gapless semiconductor. In both cases, no energy is required to excite electrons from the VB into the CB, where the excited electrons achieve 100% spin polarization at the Fermi level, as it is desirable for spintronics devices. One can flexibly tune the properties of spin gapless semiconducting materials externally by pressure, electric fields, impurities, etc^27^. Taking Fe-C_6_ doping as an example, we further investigate the effect of the dopant concentration on the band structure for a larger 5 × 5 × 1 model of MoS_2_. It is found that the gap between the spin-up VB and spin-down CB increases to 0.16 eV, indicating that the system transforms into a semiconductor. The results demonstrate that the dopant concentration is important for the character of the system, in agreement with previous findings^25^. For Fe-O_6_ doping we find 0.51 and 1.11 eV wide band gaps for the spin-up and spin-down channels, respectively. The gap between the spin-up CB and spin-down VB is 0.18 eV, so that the system retains the original semiconducting nature. For Fe-F_6_ doping the spin-up channel shows a semiconducting character with a gap of 0.99 eV, whereas the CB crosses the Fermi level in the spin-down channel, resulting in a half-metallic system. Overall, the introduction of Fe-*X*_6_ clusters in monolayer MoS_2_ can yield half-metallic and spin gapless semiconducting properties.

As the generalized gradient approximation usually underestimates the band gap of semiconductors and the spin gapless semiconducting behavior is judged from the state distribution around Fermi level, our conclusions may depend on this approximation. Hybrid functional with a certain percentage of Hartree-Fock exchange and many-body perturbation theory in the GW approximation generally lead to better agreement with experiments^26^. However, this is not a general truth but often depends on the material considered. The band gap of monolayer MoS_2_calculated in the generalized gradient approximation underestimates the experimental value of 1.8 eV by just 0.1 eV. The higher GW values of the band gap (G_0_W_0_: 2.82 eV^27^, GW: 2.97 eV^28^, 2.76 eV^29^, and GW_0_: 2.50 eV^30^) contradict experiment, whereas our calculations can give a reliable description of the electronic and magnetic properties.

**Figure 3:**
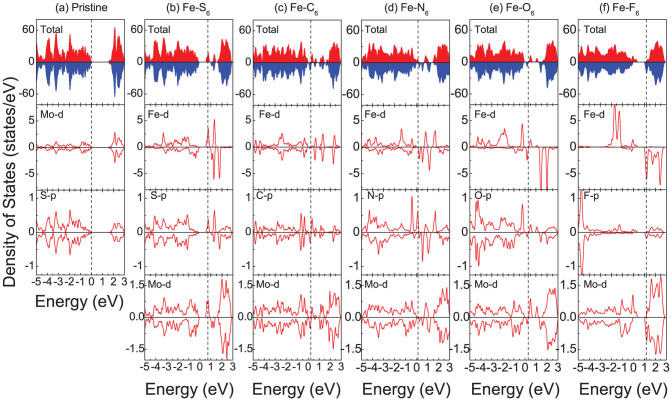
Total and projected densities of states for(a) pristine MoS_2_ as well as (b) Fe-S_6_, (c) Fe-C_6_, (d) Fe-N_6_, and (e) Fe-O_6_, and (f) Fe-F_6_ doping. The panels (b)–(f) of the 2^nd^ to 4^th^ rows refer to the Fe, nearest *X*, and nearest Mo atoms, respectively. The Fermi level is indicated by vertical dashed lines.

The density of states (DOS) of Fe-*X*_6_ doped monolayer MoS_2_ is addressed in Fig. 3. For Fe doping impurity states are formed 0.52 eV above the VB maximum and 0.09 eV below the CB minimum, reflecting *n*-doping. An increasing number of impurity states is formed when S is substituted by other *X* atoms. In the case of Fe-O_6_ doping the impurity states are close to the CB minimum (*n*-doping), as shown in Fig. 3(e), and for Fe-F_6_ doping they shift even closer (*n*-doping). When substituting S with C they shift towards the VB maximum and appear even closer for Fe-N_6_ doping for both spin channels, which is expected since the C and N atoms lack two and one electron, respectively, as compared to S. The primary contributions to the impurity states in the band gap stem from the Fe *d* states, the *p* states of the adjacent *X* atoms, and the *d* states of neighboring Mo atoms. Strong hybridization between the Fe *d* states and the *p* states of adjacent *X* atoms yields spin-polarization of the latter. In the immediate vicinity of the *X* atom only the moment of S is parallel to that of Fe, whereas those of the C, N, O, and F atoms are oriented antiparallel to Fe. Near the Fermi level we find spin splitting of the *X* *p* states, resulting in magnetic moments of 0.01, 0.07, 0.23, 0.04, and 0.01 μ_B_ per *X* atom for *X* = S, C, N O, and F, respectively, whereas the magnetic moments obtained for Fe-*X*_6_doped monolayer MoS_2_ are 1.93, 1.45, 3.18, 2.08, and 2.21 μ_B_, as shown in Table 2. The moments are mainly due to the Fe *d* orbital, with minor contributions of the *X* and neighboring Mo atoms. The Fe-*X* bond length and Fe magnetic moment increase from C to F, as the atom size decreases and the hybridization between the *X* and Fe/Mo atoms is modified.

An isolated Fe atom has a 3*d*^6^4*s*^2^electronic configuration with two additional valence electrons as compared to Mo (4*d*^5^5*s*^1^), which reflects the magnetic moment of the supercell (1.93 μ_B_). The interaction between Fe and S weakens the Mo-S bonds and induces a magnetic moment of 0.11 μ_B_ per Mo atom. The moment is smaller for Mo atoms further away. Furthermore, the four unpaired C electrons (2*s*^2^2*p*^2^ configuration) are shared with the neighboring Fe and Mo atoms, forming relatively strong bonds, as indicated by their bond lengths. The Fe *d* states show a weak spin splitting and thus a small magnetic moment. Figures 3(b)–(c) show near the Fermi level less hybridization between the C and neighboring Fe atoms, yielding a decreased magnetic moment. The smaller Mo magnetic moment is due to enhanced hybridization with C. For Fe-N_6_ doped monolayer MoS_2_ the Fe spin splitting is similar to the Fe-S_6_ system. Due to stronger Fe-N than Fe-C hybridization, the Fe spin-up states are occupied and the occupation of the spin-down states decreases. Thus, the Fe magnetic moment is slightly larger than for C doping. By weaker Mo-N than Mo-C hybridization the Mo magnetic moment is reduced. The results for Fe-O_6_ doping deviate from the above cases, while the valence electronic configuration of O (2*s*^2^2*p*^4^) is the same as for S. The Fe spin splitting is larger than for Fe-S_6_ doping, resulting in a larger magnetic moment, because the Fe-O is weaker than the Fe-S hybridization, which lowers the energy of the occupied *d* states and thus favors spin-up states. Because of stronger Mo-O hybridization, the Mo magnetic moment is slightly larger than for Fe-S_6_ doping. F (2*s*^2^2*p*^5^) has one more *p* electron than S. Weaker Fe-F than Fe-S hybridization results in enhanced Fe magnetic moments and the larger Mo magnetic moment as compared to Fe-S_6_ doping originates from weaker Mo-F hybridization. It is found that the above results are insensitive to the Fe-*X*_6_ dopant concentration.

**Figure 4:**
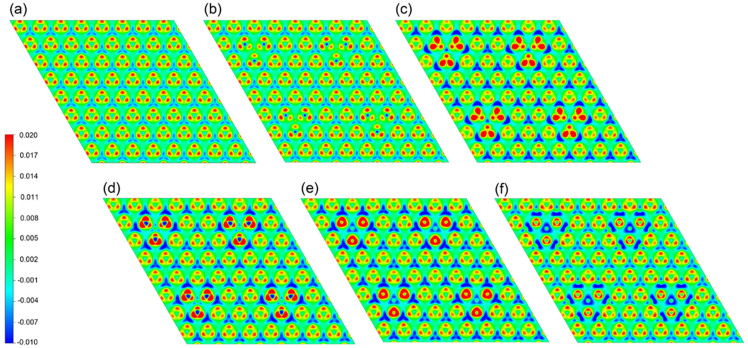
Charge density differences for (a) pristine MoS_2_ as well as (b) Fe-S_6_, (c) Fe-C_6_, (d) Fe-N_6_, (e) Fe-O_6_, and (f) Fe-F_6_ doping. The red regions represent electron accumulation and the blue regions electron depletion.

Figure 4 gives the charge density differences for Fe-*X*_6_ doped monolayer MoS_2_. The charge density difference map in Fig. 4(b) demonstrates that Fe loses less electrons than Mo. Considering that Fe has one *d* and one *s* valence electron more than Mo, Fe acts as electron donor in monolayer MoS_2_. We observe that C gains more electrons than S, despite the fact that S is more electronegative than C. The Fe atom in the Fe-C_6_ system loses more electrons than in the Fe-S_6_ system, due to the stronger bonds between the C and Fe/Mo atoms. Some extra charge is found to accumulate around the N atoms. In the case of Fe-O_6_ doping more electrons transfer from Fe and Mo to O as compared to the Fe-S_6_ system, which is consistent with the fact that O has a much higher electronegativity than S. For Fe-F_6_ doping the charge density difference is very similar to the Fe-S_6_ system far away from F, indicating that F has only a local effect on the electronic structure. More charge is transferred from Fe and Mo to F as compared to the Fe-S_6_ system. The electron density around the *X* atoms becomes more localized from C to F as the atom size decreases. Furthermore, from C to F the charge transfer from Fe/Mo to *X* is enhanced with increasing electronegativity.

## Conclusion

Our results for 4 × 4 × 1 monolayer MoS_2_ reveal the following general aspects: (1) Single Fe and Fe-F_6_ substitutions result in a half-metallic character, Fe-C_6_ and Fe-N_6_ substitutions lead to a spin gapless semiconducting behavior, and Fe-O_6_ substitution retains the original semiconducting nature. (2) Spin polarization can be induced in monolayer MoS_2_ with total magnetic moments of 1.93, 1.45, 3.18, 2.08, and 2.21 μ_B_ per 4 × 4 × 1 supercell for Fe-S_6_, Fe-C_6_, Fe-N_6_, Fe-O_6_, and Fe-F_6_ doping, respectively. (3) The magnetic moments arise mainly from the Fe atoms with small contributions from the *X* and nearest-neighbor Mo atoms, due to hybridization between the *X* *p* and Fe/Mo *d* orbital. These findings can be instrumental for the future design of MoS_2_-based electronics.

## Author Contributions

N.F. and W.M. designed the outline of the manuscript and wrote the main manuscript text. Y.C., Z.G., U.S. and H.B. contributed detailed discussions and revisions. All authors reviewed the manuscript.

## Figures and Tables

**Table 1:** Distances (Å) between the *X* and Fe (*d*_Fe-*X*_) or Mo (*d*_Mo-*X*_) atoms, and *X*-Fe-*X* bond angles (degree) for the Fe-*X*_6_ doped systems, as compared to monolayer MoS_2_

	*d*_Fe-*X*_	*d*_Mo-*X*_	*θ*_(*X*-Fe-*X*)_
pristine	—	2.41	—
Fe-S_6_	2.29	2.42	82.52
Fe-C_6_	1.93	2.04	103.03
Fe-N_6_	1.98	2.04	94.38
Fe-O_6_	2.15	2.03	92.11
Fe-F_6_	2.18	2.21	90.55

**Table 2:** Magnetic moments (μ_B_) for the Fe-*X*_6_ doped systems, as compared to monolayer MoS_2_

Magnetic moment	total	Mo	S	Fe	C	N	O	F
pristine	0	0	0	—	—	—	—	—
Fe-S_6_	1.93	0.11	0.01	1.22	—	—	—	—
Fe-C_6_	1.45	–0.05	—	0.15	0.07	—	—	—
Fe-N_6_	3.18	–0.01	—	1.03	—	0.30	—	—
Fe-O_6_	2.08	–0.20	—	3.07	—	—	0.04	—
Fe-F_6_	2.21	–0.16	—	3.34	—	—	—	0.01
